# Regulation of mitotic spindle formation by the RhoA guanine nucleotide exchange factor ARHGEF10

**DOI:** 10.1186/1471-2121-10-56

**Published:** 2009-07-28

**Authors:** Takuji Aoki, Shuji Ueda, Tohru Kataoka, Takaya Satoh

**Affiliations:** 1 Division of Molecular Biology, Department of Biochemistry and Molecular Biology, Kobe University Graduate School of Medicine, 7-5-1 Kusunoki-cho, Chuo-ku, Kobe 650-0017, Japan

## Abstract

**Background:**

The Dbl family guanine nucleotide exchange factor ARHGEF10 was originally identified as the product of the gene associated with slowed nerve-conduction velocities of peripheral nerves. However, the function of ARHGEF10 in mammalian cells is totally unknown at a molecular level. ARHGEF10 contains no distinctive functional domains except for tandem Dbl homology-pleckstrin homology and putative transmembrane domains.

**Results:**

Here we show that RhoA is a substrate for ARHGEF10. In both G1/S and M phases, ARHGEF10 was localized in the centrosome in adenocarcinoma HeLa cells. Furthermore, RNA interference-based knockdown of ARHGEF10 resulted in multipolar spindle formation in M phase. Each spindle pole seems to contain a centrosome consisting of two centrioles and the pericentriolar material. Downregulation of RhoA elicited similar phenotypes, and aberrant mitotic spindle formation following ARHGEF10 knockdown was rescued by ectopic expression of constitutively activated RhoA. Multinucleated cells were not increased upon ARHGEF10 knockdown in contrast to treatment with Y-27632, a specific pharmacological inhibitor for the RhoA effector kinase ROCK, which induced not only multipolar spindle formation, but also multinucleation. Therefore, unregulated centrosome duplication rather than aberration in cytokinesis may be responsible for ARHGEF10 knockdown-dependent multipolar spindle formation. We further isolated the kinesin-like motor protein KIF3B as a binding partner of ARHGEF10. Knockdown of KIF3B again caused multipolar spindle phenotypes. The supernumerary centrosome phenotype was also observed in S phase-arrested osteosarcoma U2OS cells when the expression of ARHGEF10, RhoA or KIF3B was abrogated by RNA interference.

**Conclusion:**

Collectively, our results suggest that a novel RhoA-dependent signaling pathway under the control of ARHGEF10 has a pivotal role in the regulation of the cell division cycle. This pathway is not involved in the regulation of cytokinesis, but instead may regulate centrosome duplication. The kinesin-like motor protein KIF3B may modulate the ARHGEF10-RhoA pathway through the binding to ARHGEF10.

## Background

Centrosomes coordinate the cytoplasmic microtubule network serving as the major microtubule-organizing centers in mammalian cells [1-3]. A single centrosome consists of a pair of centrioles surrounded by the amorphous pericentriolar material (PCM). Centrioles are cylindrical structures built of nine sets of triplet microtubules, and lie at right angles to each other and in close proximity at one end. The PCM harbors a number of protein complexes, including the γ-tubulin ring complex, which acts as a template for microtubule nucleation. During M phase of the cell cycle, two centrosomes orchestrate the assembly of bipolar mitotic spindles, which is prerequisite for accurate chromosome segregation. Aberrations in centrosome numbers, which frequently occur in aggressive human tumors, almost certainly represent one crucial cause of missegregation of chromosomes, leading to a phenotype termed genetic instability [3]. Therefore, the rigorous control of the duplication cycle of the centrosome is vital for the cell. However, our understanding of the regulation of the centrosome cycle remains incomplete.

The signal transducing GTPase RhoA has been implicated in a variety of cellular responses in mammalian cells, being regulated by Dbl family guanine nucleotide exchange factors (GEFs) in a cell type- and signal-specific manner [4]. Dbl Family GEFs contain Dbl homology (DH) and pleckstrin homology (PH) domains in tandem, which are responsible for enhancement of guanine nucleotide exchange on the target GTPase. The DH domain is a catalytic domain whereas the PH domain is thought to be required for the modulation of protein conformation and subcellular localization [4]. A well characterized function of RhoA is the regulation of cell division, particularly cytokinesis [5,6]. Upon onset of anaphase, the GEF ECT2 activates RhoA, leading to the accumulation of activated RhoA at the cleavage furrow. Activated RhoA in turn directs assembly and contraction of the contractile ring through the activation of various downstream effectors, including formin family proteins, the Rho-dependent kinase ROCK and citron kinase. Formin family proteins regulate actin polymerization, allowing the formation of the contractile ring. ROCK and citron kinase are involved in the control of contractile ring ingression and completion of cytokinesis, respectively, through phosphorylation of regulatory myosin light chains.

Increasing evidence has emerged that RhoA plays a pivotal role not only in cytokinesis, but also in mitosis [6]. For instance, ROCK regulates mother centriole movement in late telophase and exit from mitosis [7]. During prophase/prometaphase, RhoA and its GEF Lfc are required for the assembly of the mitotic spindle [8]. Separation and positioning of duplicated centrosomes after nuclear envelope breakdown, which are important for correct spindle alignment, may also be regulated by RhoA and ROCK [9].

**Figure 1:**
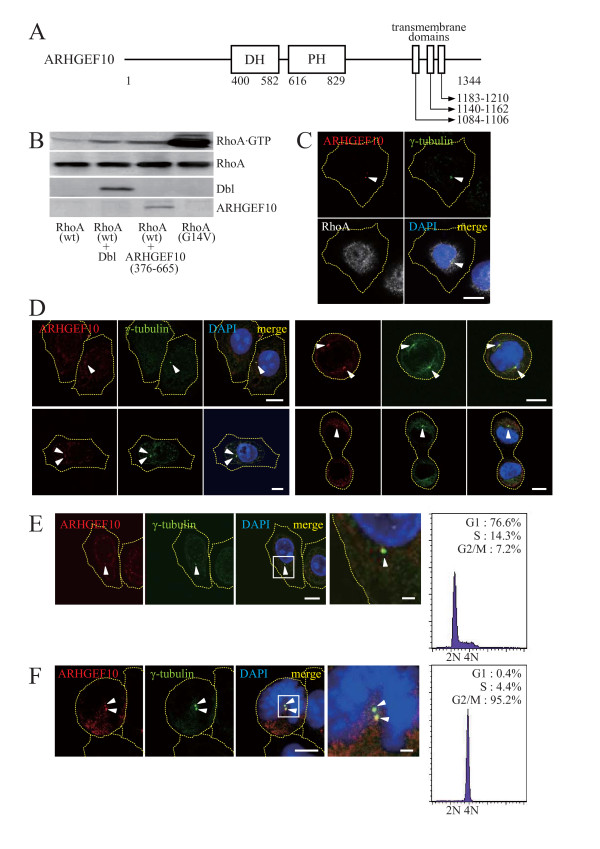
**Substrate specificity and subcellular localization of ARHGEF10**. (A) The domain structure of ARHGEF10. Amino acid residue numbers are shown. DH, Dbl homology; PH, pleckstrin homology. (B) GEF activity of ARHGEF10 toward RhoA. HA × 3-tagged wild-type (wt) or constitutively activated (G14V) RhoA was expressed with Myc-tagged Dbl or Myc-tagged ARHGEF10(376–665) in COS7 cells. Levels of the GTP-bound form were measured by pull-down assays using GST-Dia(1–304). Expression levels of RhoA and GEFs were determined by immunostaining with anti-tag antibodies. (C) Co-localization of ARHGEF10 and RhoA in centrosomes. HA × 3-tagged RhoA(wt) was expressed in HeLa cells, and indicated proteins were immunostained. Scale bar, 10 μm. (D) Subcellular localization of ARHGEF10 in unsynchronized cells. HeLa cells were cultivated in the growth medium, and indicated proteins were immunostained. Scale bar, 10 μm. (E) Subcellular localization of ARHGEF10 in G1/S phase. HeLa cells were synchronized in G1/S phase with thymidine, and indicated proteins were immunostained. The high magnification image of the boxed area in the 3rd panel is shown in the 4th panel. Scale bar, 10 μm (1st-3rd panels) and 2 μm (the 4th panel). Flow cytometric analysis of DNA content is also shown. (F) Subcellular localization of ARHGEF10 in M phase. HeLa cells were synchronized in M phase with nocodazole, and indicated proteins were immunostained. The high magnification image of the boxed area in the 3rd panel is shown in the 4th panel. Scale bar, 10 μm (1st-3rd panels) and 2 μm (the 4th panel). Flow cytometric analysis of DNA content is also shown. In (C) to (F), nuclei were stained with 4',6-diamidino-2-phenylindole (DAPI), and arrowheads indicate centrosomes. Thin dotted lines in cell images in (C) to (F) indicate the contour of the cell.

The Dbl family GEF ARHGEF10 was originally identified as the product of the gene associated with slowed nerve-conduction velocities of peripheral nerves [10]. However, underlying molecular mechanisms are totally unknown. Also, the function of ARHGEF10 in mammalian cell cycle progression remains to be clarified. ARHGEF10 was initially reported to possess a DH domain in its N-terminal portion, but lack the PH domain [11]. Recently, however, a less conserved PH domain in tandem with the DH domain was identified [12] (see Figure 1A). C-terminally located two transmembrane domains have also been predicted from amino acid sequence [11]. Based on the similarity between human and mouse sequences, we identified an additional putative transmembrane domain, which lies immediately adjacent and C-terminal to the original ones (Figure 1A). Except for the pair of DH and PH domains and putative transmembrane domains, no functional domain structures have been noted for ARHGEF10. Herein, we investigate the role of ARHGEF10 in cell division control and demonstrate that ARHGEF10 regulates the centrosome number through the action on its target RhoA.

## Methods

### Antibodies

Antibodies against Myc (mouse monoclonal [sc-40]), FLAG (mouse monoclonal [F3165] and rabbit polyclonal [F7425]) and hemagglutinin (HA) (rat monoclonal [1867423]) epitope tags were purchased from Santa Cruz Biotechnology, Sigma-Aldrich and Roche Applied Science, respectively. An antibody against ARHGEF10 (mouse polyclonal [H00009639-B01]) was purchased from Abnova. Antibodies against α-tubulin (mouse monoclonal [T6199]), β-tubulin (mouse monoclonal [T0198]), γ-tubulin (mouse monoclonal [T6557], rabbit polyclonal [T3320]) and centrin (rabbit polyclonal [C7736]) were purchased from Sigma-Aldrich. An antibody against pericentrin (rabbit polyclonal [PRB-432C]) was purchased from Covance. Antibodies against Aurora-A (rabbit monoclonal [#4718]) and centrin-2 (rabbit monoclonal [#2091]) were purchased from Cell Signaling Technology. Anti-mouse IgG, anti-rabbit IgG and anti-rat IgG antibodies conjugated with Alexa Fluor 488/546/647 were purchased from Invitrogen (Molecular Probes).

### Plasmids, small interfering RNAs (siRNAs) and polymerase chain reaction (PCR) primers

cDNAs encoding HA × 3-tagged human small GTPases (RhoA [wt], RhoA [G14V], Rac1 [G12V] and Cdc42 [G12V]) were subcloned into pEF-BOS for expression in mammalian cells [13]. cDNAs encoding N-terminally Myc-tagged full-length and truncated (amino acids 376–665 containing the DH domain) ARHGEF10 (also termed KIAA0294, NCBI accession number NM_014629) were subcloned into the mammalian expression vector pCMV5 (NCBI accession number AF239249). The cDNA encoding N-terminally FLAG-tagged KIF3B (also termed KIAA0359, NCBI accession number AB002357) was subcloned into the mammalian expression vector pCMV5. pCMV5-Myc-Dbl-I [14] and pFLAG-CMV2-EGFP [15] were described previously. siRNAs for ARHGEF10 (Stealth select HSS114404 [#1] and Stealth select HSS114405 [#2]), RhoA (Stealth select HSS100654 [#1], Stealth select HSS100655 [#2] and Stealth select HSS100656 [#3]) and KIF3B (Stealth select HSS113902 [#1], Stealth select HSS113903 [#2] and Stealth select HSS113904 [#3]) were purchased from Invitrogen. The control siRNA (Stealth RNAi negative control medium GC duplex #3) was also purchased from Invitrogen. Reverse transcriptase (RT)-PCR was performed using specific primers (5'-caatatgagaagccgctgtc-3' and 5'-catattcacggtatgcatccag-3' for ARHGEF10 [35 cycles], 5'-gatatcgaggtggatggaaag-3' and 5'-cacatcagtataacatcggtatc-3' for RhoA [32 cycles], 5'-ggaaactacatcctataaccag-3' and 5'-cctgtatctcatcttcatcctg-3' for KIF3B [35 cycles] and 5'-ccccttcattgacctcaactac-3' and 5'-atgaccttgcccacagccttgg-3' for glyceraldehyde-3-phosphate dehydrogenase (GAPDH) [35 cycles]) as described [13].

### Cell culture, transfection and synchronization

HeLa, COS7 and U2OS cells were cultivated in Dulbecco's modified Eagle's Medium (Nacalai Tesque, Japan) supplemented with 10% (v/v) fetal bovine serum, 100 IU/ml penicillin and 100 μg/ml streptomycin. For transfection, SuperFect (Qiagen, for plasmids only), Lipofectamine RNAiMAX (Invitrogen, for siRNAs only) and Lipofectamine 2000 (Invitrogen, for a mixture of plasmids and siRNAs) were used according to the manufacturer's instructions (24 h-incubation with cells).

Synchronization of HeLa cells in M phase was achieved by incubation of cells in 100 ng/ml nocodazole (Sigma-Aldrich) for 20 h. Mitotic cells were collected by mitotic shake-off, and released from the nocodazole block. If not specified, cells were fixed, and subjected to immunofluorescent staining after 90 min-incubation without nocodazole. In some experiments, cells were pretreated with 50 μM Y-27632 (Calbiochem), which was also included during nocodazole treatment.

Synchronization of HeLa cells in G1/S phase was achieved by a double thymidine block. Cells were incubated in 10 mM thymidine (Sigma-Aldrich) for 24 h, and then released from the first thymidine block for 10 h in the culture medium. A second block was initiated by adding 10 mM thymidine, and cells were maintained for 14 h. Cells were then released from the thymidine block for the indicated times.

Synchronization of U2OS cells in S phase was achieved by incubation of cells in 1.6 μg/ml aphidicolin (Sigma-Aldrich) for 16 h.

### Immunofluorescent microscopy

Cells were fixed with cold methanol for 10 min, and immunofluorescent staining was performed as previously described [13-15] Images were obtained using a confocal laser-scanning microscope (LSM 510 META; Carl Zeiss) and processed by the Zeiss LSM Image Browser, version 3.5.

### Pull-down assays

Pull-down assays for the activated form of RhoA was carried out as described previously [13].

### Immunoprecipitation and immunoblotting

COS7 cells were dissolved in lysis buffer (50 mM Tris-HCl [pH 7.4], 150 mM NaCl, 20 mM MgCl_2_, 0.5% [v/v] Nonidet P-40), and centrifuged at 20,000 × *g *for 10 min at 4°C. Cell lysates were incubated with protein G-Sepharose beads and a specific antibody for 30 min at 4°C. Subsequently, protein G-Sepharose beads were washed three times with lysis buffer, and precipitated proteins were subjected to SDS-PAGE and immunoblotting. Immunoblotting was performed as previously described[15].

### Flow cytometric analysis of DNA content

Growing or synchronized HeLa cells were fixed with ice-cold 70% [v/v] ethanol, and then resuspended in phosphate-buffered saline containing 20 μg/ml ribonuclease A (Sigma). DNA was stained with 20 μg/ml propidium iodide (Dojindo), and cytometric analysis was performed on a flow cytometer (FACScan, Becton-Dickinson Immunocytometry Systems). Collected data were processed by CELLQuest.

### Yeast two-hybrid screening

A MATCH MAKER yeast two-hybrid human brain library (HY4004AH, Clontech, 5 × 10^6 ^independent clones) was screened using an N-terminal region (amino acids 1–1083) of ARHGEF10 as a bait according to the manufacturer's instructions. The interaction between isolated clones and ARHGEF10(1–1083) was confirmed by growth on the selection plate (synthetic dropout medium-Trp/-Leu/-His/-Ade/+2.5 mM 3-aminotriazole) and the β-galactosidase assay according to the manufacturer's instructions.

## Results

### Involvement of ARHGEF10 in the regulation of mitotic spindle formation

As a first step to clarify the function of ARHGEF10 at a molecular level, we attempted to identify substrate Rho family GTPases for putative GEF activity of ARHGEF10 by a pull-down assay (Figure 1B). In this assay, the activated form of the GTPase accumulated within the cell was precipitated by a probe that selectively recognizes the active form, and visualized by immunoblotting. When co-expressed with a fragment of ARHGEF10 that contains catalytic DH and regulatory PH domains, the activated form of ectopically expressed wild-type RhoA was increased, indicating that ARHGEF10 actually showed GEF activity toward RhoA. GEF activity of ARHGEF10 was comparable to that of Dbl, a previously characterized GEF for RhoA. In contrast, GTP loading to Rac1 and Cdc42 was not enhanced by ARHGEF10 as previously described [[12] and data not shown]. Although a previous study demonstrated GEF activity of ARHGEF10 toward RhoB, we were unable to detect significant GEF activity toward RhoB [[12] and data not shown]. When ectopically expressed in HeLa cells, a subset of RhoA was indeed co-localized with ARHGEF10 in centrosomes (Figure 1C) (see below).

Subcellular localization of ARHGEF10 was then determined by immunofluorescent staining (Figures 1D–1F). Of note, ARHGEF10 was localized, at least in part, in centrosomes visualized by counter staining of γ-tubulin in unsynchronized HeLa cells in various phases of the cell cycle (Figure 1D). Such centrosomal localization of ARHGEF10 was also evident at least in G1/S and M phases as demonstrated by using synchronized cell culture (Figures 1E and 1F). Virtually all centrosomes that we detected by γ-tubulin staining were also positive for ARHGEF10. Synchronization of these cells was confirmed by flow cytometric analysis of DNA content (Figures 1E and 1F). Therefore, ARHGEF10 may be involved in the control of the cell division cycle.

**Figure 2:**
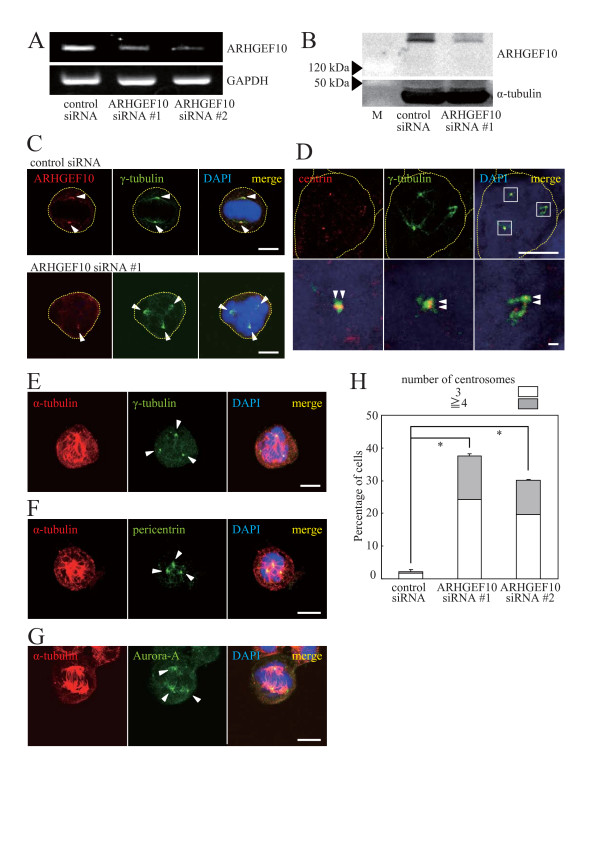
**Multipolar spindle formation in ARHGEF10-knockdown HeLa cells**. (A) Effect of ARHGEF10 siRNAs on mRNA expression. Expression levels of endogenous ARHGEF10 and GAPDH (control) mRNAs in HeLa cells transfected with indicated siRNAs were measured by RT-PCR. (B) Effect of ARHGEF10 siRNA #1 on protein expression. Expression levels of Myc-tagged ARHGEF10 and α-tubulin (control) in HeLa cells transfected with indicated siRNAs were measured by immunoblotting. M, molecular weight markers. (C) Subcellular localization of ARHGEF10 in M phase. HeLa cells were transfected with ARHGEF10 siRNA #1 or control siRNA and synchronized in M phase with nocodazole. Indicated proteins were immunostained. Scale bar, 10 μm. (D) Immunostaining of centrin and γ-tubulin in ARHGEF10-knockdown cells. HeLa cells were transfected with ARHGEF10 siRNA #1 and synchronized in M phase with nocodazole. Indicated proteins were immunostained. High magnification images of boxed areas are shown in lower panels. Scale bar, 10 μm (upper panels) and 1 μm (lower panels). (E) Immunostaining of α-tubulin and γ-tubulin in ARHGEF10-knockdown cells. Indicated proteins in HeLa cells treated as in (D) were immunostained. Scale bar, 10 μm. (F) Immunostaining of α-tubulin and pericentrin in ARHGEF10-knockdown cells. Indicated proteins in HeLa cells treated as in (D) were immunostained. Scale bar, 10 μm. (G) Immunostaining of α-tubulin and Aurora-A in ARHGEF10-knockdown cells. Indicated proteins in HeLa cells treated as in (D) were immunostained. Scale bar, 10 μm. (H) Quantification of multipolar spindle formation in ARHGEF10-knockdown cells. HeLa cells were transfected with indicated siRNAs and synchronized in M phase with nocodazole. The percentage of cells containing multiple centrosomes are shown as the means ± S.E. for three independent experiments. *, *P *< 0.01. In (C) to (G), nuclei were stained with DAPI, and arrowheads indicate centrosomes (C, E, F and G) or centrioles (D). Thin dotted lines in cell images in (C) and (D) indicate the contour of the cell.

To gain further insights into the function of ARHGEF10 during cell cycle progression, expression of endogenous ARHGEF10 in HeLa cells was downregulated by two different sets of siRNAs. Decrease in the mRNA level was confirmed by RT-PCR analysis (Figure 2A), and the effect on protein expression was assessed by the use of an epitope-tagged ectopically expressed ARHGEF10 because we were unable to detect endogenous ARHGEF10 by immunoblotting (Figure 2B). Following synchronization in M phase by nocodazole treatment, aberrant multipolar spindle formation was observed in 30~40% of ARHGEF10-knockdown cells whereas more than 95% of control siRNA-transfected cells showed normal bipolar spindles under the same culture conditions (Figures 2C–2H). This aberrant spindle formation was not merely due to nocodazole treatment because G2/M arrest of control siRNA-transfected cells by nocodazole did not induce multipolar spindles (Figures 2C and 2H), and we actually detected multiple centrosomes in mitotic ARHGEF10-knockdown cells without nocodazole treatment (data not shown). ARHGEF10-specific siRNA indeed reduced the amount of centrosome-localized ARHGEF10 (Figure 2C). Interestingly, we reproducibly observed that two centrosomes contain markedly reduced amounts of ARHGEF10 whereas the expression level of ARHGEF10 in the other centrosome was decreased only partly. All mitotic spindle poles in ARHGEF10-knockdown cells appeared to consist of two centrioles as shown by immunostaining for the centriole marker centrin (Figure 2D). Each centrosome was indeed positive for PCM markers γ-tubulin, pericentrin and Aurora-A (Figures 2E–2G).

### RhoA regulates mitotic spindle formation downstream of ARHGEF10

**Figure 3:**
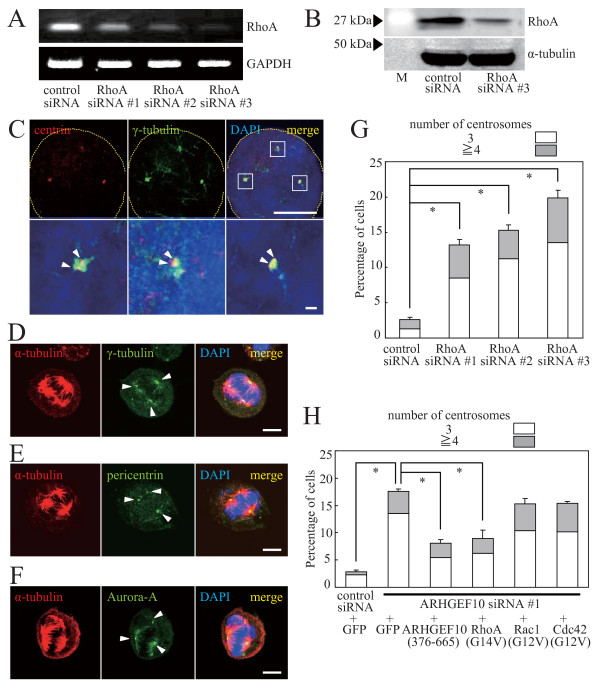
**Role of RhoA downstream of ARHGEF10**. (A) Effect of RhoA siRNAs on mRNA expression. Expression levels of endogenous RhoA and GAPDH (control) mRNAs in HeLa cells transfected with indicated siRNAs were measured by RT-PCR. (B) Effect of RhoA siRNA #3 on protein expression. Expression levels of HA × 3-tagged RhoA(wt) and α-tubulin (control) in HeLa cells transfected with indicated siRNAs were measured by immunoblotting. M, molecular weight markers. (C) Immunostaining of centrin and γ-tubulin in RhoA-knockdown cells. HeLa cells were transfected with RhoA siRNA #2 and synchronized in M phase with nocodazole. Indicated proteins were immunostained. High magnification images of boxed areas are shown in lower panels. Scale bar, 10 μm (upper panels) and 1 μm (lower panels). (D) Immunostaining of α-tubulin and γ-tubulin in RhoA-knockdown cells. Indicated proteins in HeLa cells treated as in (C) were immunostained. Scale bar, 10 μm. (E) Immunostaining of α-tubulin and pericentrin in RhoA-knockdown cells. Indicated proteins in HeLa cells treated as in (C) were immunostained. Scale bar, 10 μm. (F) Immunostaining of α-tubulin and Aurora-A in RhoA-knockdown cells. Indicated proteins in HeLa cells treated as in (C) were immunostained. Scale bar, 10 μm. (G) Quantification of multipolar spindle formation in RhoA-knockdown cells. HeLa cells were transfected with indicated siRNAs and synchronized in M phase with nocodazole. The percentage of cells containing multiple centrosomes are shown as the means ± S.E. for three independent experiments. *, *P *< 0.01. (H) Suppression of multipolar spindle formation in ARHGEF10-knockdown cells by RhoA. HeLa cells transfected with ARHGEF10 siRNA #1 or control siRNA in combination with an expression plasmid for green fluorescent protein (GFP) (control), Myc-tagged ARHGEF10(376–665) or HA × 3-tagged constitutively activated GTPases were synchronized in M phase with nocodazole. The percentage of cells containing multiple centrosomes are shown as the means ± S.E. for three independent experiments. *, *P *< 0.01. In (C) to (F), nuclei were stained with DAPI, and arrowheads indicate centrioles (C) or centrosomes (D, E and F). Thin dotted lines in cell images in (C) indicate the contour of the cell.

We next examined the involvement of Rho family GTPases in ARHGEF10-dependent regulation of mitotic spindle formation. Expression of the ARHGEF10 target RhoA was reduced by specific siRNA sets (Figures 3A and 3B), and the effect on spindle pole formation was examined. Similarly to ARHGEF10 knockdown, multiple spindle poles emerged (Figures 3C–3G). Each spindle pole in fact harbored one centrosome consisting of two centrioles and the PCM as shown by staining of marker proteins (Figures 3C–3F). We also tried to see the effect of C3 exoenzyme, which specifically inactivates Rho proteins, without success due to extensive cell death upon synchronization of the cell (data not shown). Furthermore, multipolar spindle formation induced by knockdown of ARHGEF10 was rescued by ectopic expression of a constitutively activated mutant of RhoA, but not Rac1 and Cdc42, suggesting the involvement of RhoA downstream of ARHGEF10 (Figure 3H). Rescue by constitutively activated RhoA was incomplete, but comparable to that by the ARHGEF10 siRNA #1-resistant catalytic DH domain of ARHGEF10 (ARHGEF10 [376–665]). The percentage of cells containing multiple mitotic spindle poles shown in Figure 3H was lower compared to that in Figure 2H because the amount of ARHGEF10 siRNA was reduced when introduced with plasmid DNAs.

### Multipolar spindle formation in ARHGEF10-knockdown cells is not ascribed to defects in cytokinesis

**Figure 4:**
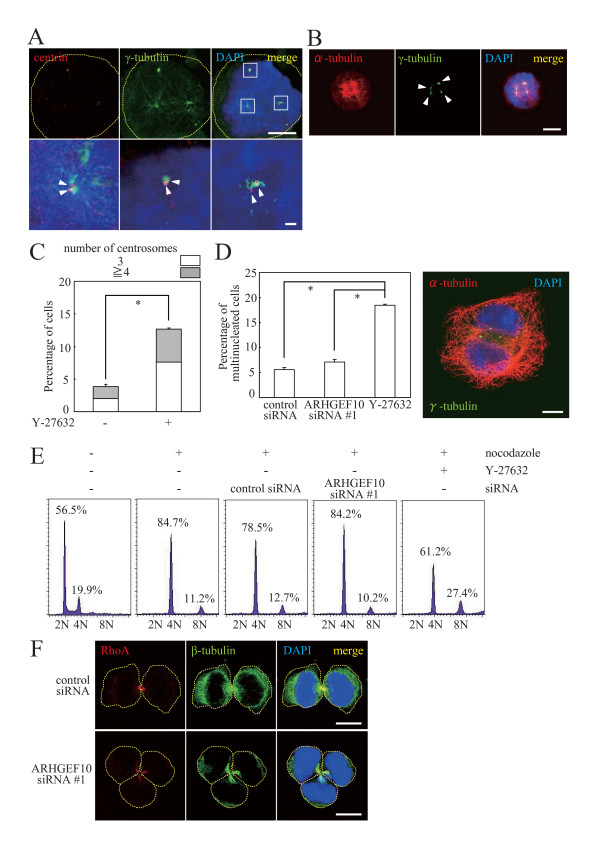
**Multinucleation in Y-27632-treated, but not ARHGEF10-knockdown, HeLa cells**. (A) Immunostaining of centrin and γ-tubulin in Y-27632-treated cells. HeLa cells were treated with Y-27632 for 4 h and synchronized in M phase with nocodazole. Indicated proteins were immunostained. High magnification images of boxed areas are shown in lower panels. Scale bar, 10 μm (upper panels) and 1 μm (lower panels). (B) Immunostaining of α-tubulin and γ-tubulin in Y-27632-treated cells. Indicated proteins in HeLa cells treated as in (A) were immunostained. Scale bar, 10 μm. (C) Quantification of multipolar spindle formation in Y-27632-treated cells. HeLa cells were treated with Y-27632 for 4 h and synchronized in M phase with nocodazole. The percentage of cells containing multiple centrosomes are shown as the means ± S.E. for three independent experiments. *, *P *< 0.01. (D) Increase of multinucleated cells upon Y-27632 treatment, but not ARHGEF10 knockdown. HeLa cells were transfected with ARHGEF10 siRNA #1 or treated with Y-27632 for 48 h and synchronized in M phase with nocodazole. Multinucleated cells as shown in the right panel were counted after 4 h-incubation without nocodazole. The percentage of multinucleated cells are shown as the means ± S.E. for three independent experiments. *, *P *< 0.01. Scale bar, 10 μm. (E) Flow cytometric analysis of DNA content in ARHGEF10-knockdown and Y-27632-treated HeLa cells. Representative fluorescence-activated cell sorter histograms and the percentage of cells contained in each peak are shown. (F) Cytokinesis of ARHGEF10-knockdown cells. HeLa cells were transfected with ARHGEF10 siRNA #1 or control siRNA in combination with an expression plasmid for HA × 3-tagged RhoA(wt) and synchronized in M phase with nocodazole. Indicated proteins were immunostained after 3 h-incubation without nocodazole. Representative cells during cytokinesis are shown. Scale bar, 10 μm. In (A), (B), (D) and (F), nuclei were stained with DAPI, and arrowheads indicate centrioles (A) or centrosomes (B). Thin dotted lines in cell images in (A) and (F) indicate the contour of the cell.

It is important to clarify whether multiple spindle formation in ARHGEF10-knockdown cells was due to defects in cytokinesis. To this end, we utilized the ROCK pharmacological inhibitor Y-27632 as a control because ROCK is intimately involved in the control of cytokinesis downstream of RhoA [5,6]. As a first step, mitotic spindle formation in Y-27632-treated cells was examined under conditions similar to those for siRNA-mediated knockdown of ARHGEF10. HeLa cells were treated with Y-27632 for 4 h prior to challenge with nocodazole, and then subjected to synchronization in M phase. Y-27632 induced multiple mitotic spindle formation resembling that observed in ARHGEF10- or RhoA-knockdown cells (Figures 4A–4C), which may be ascribed to aberration in cytokinesis. The percentage of multinucleated cells was indeed increased upon Y-27632 treatment whereas virtually no increase was observed in ARHGEF10-knockdown cells (Figure 4D). Fluorescence-activated cell sorter analysis also support the notion that Y-27632 treatment, but not ARHGEF10 knockdown, caused multinucleation presumably due to defects incytokinesis.

Nocodazole treatment increased 4N and 8N cell populations as expected (4N, 84.3 ± 0.809% [for three independent experiments]; 8N, 11.2 ± 0.866% [for three independent experiments]). Transfection of control (4N, 79.4 ± 2.46% [for three independent experiments]; 8N, 11.1 ± 0.819% [for three independent experiments]) or ARHGEF10-specific #1 (4N, 86.9 ± 1.88% [for three independent experiments]; 8N, 7.27 ± 1.48% [for three independent experiments]) siRNA did not significantly affect the percentage of multi-nucleated (8N) cells. In contrast, Y27632-treated cells showed higher percentage of multi-nucleated (8N) cells (4N, 60.5 ± 5.55% [for three independent experiments]; 8N, 24.9 ± 2.17% [for three independent experiments]). (Figure 4E). Cleavage furrow formation and cytokinesis were indeed observed in ARHGEF10-knockdown tripolar cells(Figure 4F). In addition, RhoA was indeed accumulated in the midbody in ARHGEF10-knockdown tripolar as well as control cells(Figure 4F). Overall, these results suggest that occurrence of multiple centrosomes in ARHGEF10-knockdown cells may not be due to abnormalities incytokinesis. Instead, ARHGEF10 may have a crucial role in the regulation of centrosome duplication considering that ARHGEF10 is localized in centrosomes (Figures 1D–F). If this is the case, aberrant centrosome duplication may occur causing the formation of multiple spindle poles when ARHGEF10 was knocked down.

### The kinesin-like motor protein KIF3B may regulate ARHGEF10 as a binding partner

**Figure 5:**
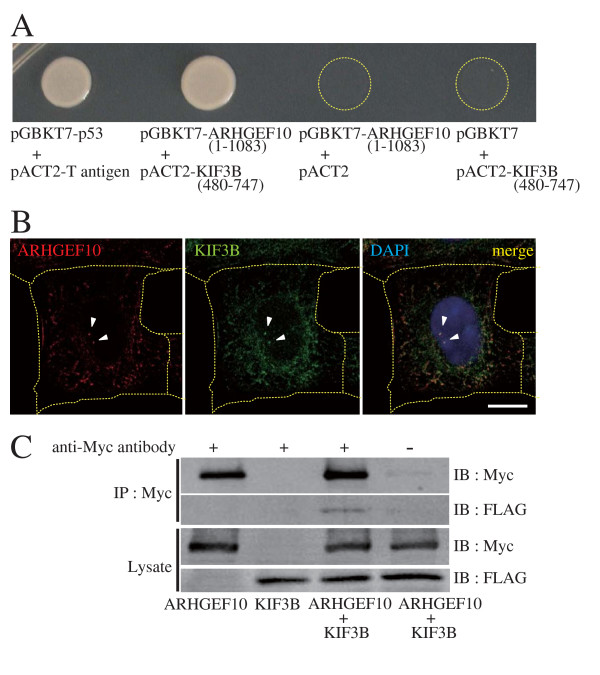
**Interaction of KIF3B with ARHGEF10**. (A) Yeast two-hybrid interaction of KIF3B with ARHGEF10. cDNAs for ARHGEF10(1–1083) and KIF3B(480–747) were subcloned into yeast two-hybrid vectors pGBKT7 and pACT2, respectively, and two-hybrid interaction was assessed by growth on the selection plate (synthetic dropout medium-Trp/-Leu/-His/-Ade/+2.5 mM 3-aminotriazole). pGBKT7-p53 and pACT2-T antigen were used as a positive control. (B) Co-localization of ARHGEF10 and KIF3B in HeLa cells. HeLa cells were transfected with an expression plasmid for FLAG-tagged KIF3B, and indicated proteins were immunostained. Arrowheads indicate centrosomes. Scale bar, 10 μm. Thin dotted lines indicate the contour of the cell. (C) Co-immunoprecipitation of KIF3B with ARHGEF10. Myc-tagged ARHGEF10 and FLAG-tagged KIF3B were expressed in COS7 cells as indicated, and association of these proteins was examined by co-immunoprecipitation. IP, immunoprecipitation; IB, immunoblotting.

**Figure 6:**
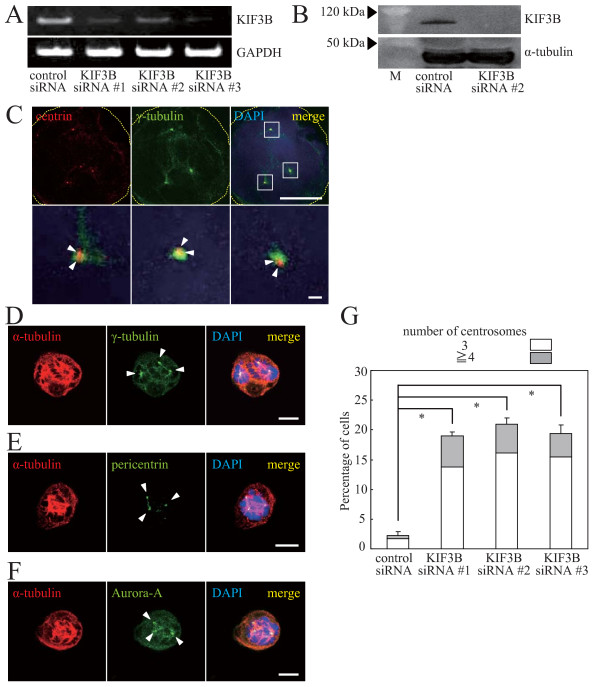
**Multipolar spindle formation in KIF3B-knockdown HeLa cells**. (A) Effect of KIF3B siRNAs on mRNA expression. Expression levels of endogenous KIF3B and GAPDH (control) mRNAs in HeLa cells transfected with indicated siRNAs were measured by RT-PCR. (B) Effect of KIF3B siRNA #2 on protein expression. Expression levels of FLAG-tagged KIF3B and α-tubulin (control) in HeLa cells transfected with indicated siRNAs were measured by immunoblotting. M, molecular weight markers. (C) Immunostaining of centrin and γ-tubulin in KIF3B-knockdown cells. HeLa cells were transfected with KIF3B siRNA #2 and synchronized in M phase with nocodazole. Indicated proteins were immunostained. High magnification images of boxed areas are shown in lower panels. Scale bar, 10 μm (upper panels) and 1 μm (lower panels). (D) Immunostaining of α-tubulin and γ-tubulin in KIF3B-knockdown cells. Indicated proteins in HeLa cells treated as in (C) were immunostained. Scale bar, 10 μm. (E) Immunostaining of α-tubulin and pericentrin in KIF3B-knockdown cells. Indicated proteins in HeLa cells treated as in (C) were immunostained. Scale bar, 10 μm. (F) Immunostaining of α-tubulin and Aurora-A in KIF3B-knockdown cells. Indicated proteins in HeLa cells treated as in (C) were immunostained. Scale bar, 10 μm. (G) Quantification of multipolar spindle formation in KIF3B-knockdown cells. HeLa cells were transfected with indicated siRNAs and synchronized in M phase with nocodazole. The percentage of cells containing multiple centrosomes are shown as the means ± S.E. for three independent experiments. *, *P *< 0.01. In (C) to (F), nuclei were stained with DAPI, and arrowheads indicate centrioles (C) or centrosomes (D, E and F). Thin dotted lines in cell images in (C) indicate the contour of the cell.

Through yeast two-hybrid screening for ARHGEF10-interacting proteins, we isolated a clone encoding the C-terminal portion (amino acids 480–747) of the kinesin-like motor protein KIF3B. This portion does not overlap with the microtubule-binding motor domain. The interaction was confirmed by growth on the selection plate (Figure 5A) and the β-galactosidase assay (data not shown). ARHGEF10 and KIF3B indeed co-localized in centrosomes in HeLa cells (Figure 5B). The interaction between ARHGEF10 and KIF3B was further confirmed by co-immunoprecipitation from COS7 cells (Figure 5C). Intriguingly, siRNA-mediated knockdown of KIF3B also induced multiple spindle poles (Figure 6). Each spindle pole indeed contains two centrioles and the PCM similarly to ARHGEF10- or RhoA-knockdown cells (Figures 6C–6G). Thus, KIF3B may exert a regulatory role in ARHGEF10-RhoA-mediated control of mitotic spindle formation.

### The supernumerary centrosome phenotype was also observed in S phase-arrested osteosarcoma U2OS cells when the expression of ARHGEF10, RhoA or KIF3B was reduced

**Figure 7:**
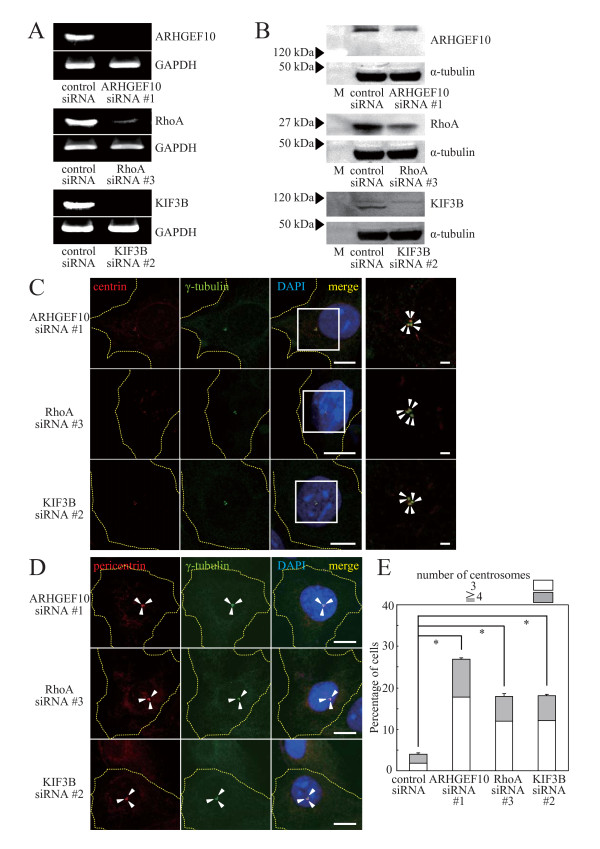
**Supernumerary centrosomes in aphidicolin-treated U2OS cells**. (A) Effect of siRNAs for ARHGEF10, RhoA and KIF3B on mRNA expression. Expression levels of ARHGEF10, RhoA, KIF3B and GAPDH (control) mRNAs in U2OS cells transfected with indicated siRNAs were measured by RT-PCR. (B) Effect of siRNAs for ARHGEF10, RhoA and KIF3B on protein expression. Expression levels of Myc-tagged ARHGEF10, HA × 3-tagged RhoA(wt), FLAG-tagged KIF3B and α-tubulin (control) in U2OS cells transfected with indicated siRNAs were measured by immunoblotting. M, molecular weight markers. (C) Immunostaining of centrin and γ-tubulin in ARHGEF10-, RhoA- and KIF3B-knockdown cells. U2OS cells were transfected with ARHGEF10 siRNA #1, RhoA siRNA #3 or KIF3B siRNA #2 and synchronized in S phase with aphidicolin. Indicated proteins were immunostained. High magnification images of boxed areas are shown in right panels. Scale bar, 2 μm (right panels) and 10 μm (other panels). (D) Immunostaining of pericentrin and γ-tubulin in ARHGEF10-, RhoA- and KIF3B-knockdown cells. Indicated proteins in U2OS cells treated as in (C) were immunostained. Scale bar, 10 μm. (E) Quantification of supernumerary centrosomes in ARHGEF10-, RhoA- and KIF3B-knockdown cells. U2OS cells were transfected with indicated siRNAs and synchronized in S phase with aphidicolin. The percentage of cells containing multiple centrosomes are shown as the means ± S.E. for three independent experiments. *, *P *< 0.01. In (C) and (D), nuclei were stained with DAPI, and arrowheads indicate centrioles (C) or centrosomes (D). Thin dotted lines in cell images in (C) and (D) indicate the contour of the cell.

The effect of knockdown of ARHGEF10, RhoA or KIF3B was also examined in osteosarcoma U2OS cells arrested in S phase by aphidicolin treatment (Figure 7). Supernumerary centrosomes containing a pair of centrioles and the PCM were observed in ARHGEF10-, RhoA- or KIF3B-knockdown cells (Figure 7C–E).

However, when the expression of ARHGEF10, RhoA or KIF3B was reduced, enhanced formation of multiple procentrioles surrounding a parental centriole as reported in Plk4-overexpressing cells [16] was not observed (data not shown).

## Discussion

In mitotic ARHGEF10-knockdown cells, we observed multiple spindle poles, each of which contains two centrioles and the PCM. One possible mechanism for the formation of such supernumerary centrosomes is aborted cell division [1-3]. Aneuploid cells generated through aberrant mitotic exit contain more than two centrosomes, which may form multipolar spindles in next M phase. Initially, we thought that aberrant division of cells lacking ARHGEF10 may frequently occur because RhoA is known to be involved in many facets of mitotic and cytokinetic control. However, the occurrence of multinucleated cells was not affected by ARHGEF10 knockdown in contrast to treatment with a ROCK inhibitor, which significantly enhanced the formation of multinucleated cells (Figure 4). Furthermore, normal ingression of the cleavage furrow was, in fact, detected in ARHGEF10-knockdown cells as well (Figure 4F). Therefore, it is not feasible that ARHGEF10 is responsible for RhoA activation in mitosis and cytokinesis. Instead, ARHGEF10 may regulate RhoA during interphase.

Deregulated centrosome duplication also leads to the formation of multiple centrosomes and multipolar spindles [1-3]. Thus, the multi-centrosome phenotype observed in ARHGEF10-knockdown cells may be ascribed to disorder in centrosome duplication. Localization of ARHGEF10 in centrosomes strongly supports this notion. Centriole duplication is initiated by the activation of cyclin-dependent kinase 2 around the G1/S transition, concurrent with DNA replication, and short daughter centrioles elongate during S and G2 phases [17-19]. Following completion of centriole elongation at the G2/M transition, centrosome maturation and separation occur, with a pair of centrioles in each centrosome. Centrosome duplication must occur precisely once every cell cycle, being inextricably coupled with other cell cycle-dependent events. To ensure this, cells are afforded mechanisms (1) to limit centriole duplication to once in every cell division cycle and (2) to limit the number of progenitor centrioles to one per pre-existingcentriole.

Plenty of molecules that positively regulate centrosome duplication, including Polo-like kinase-4 [16], cyclin-dependent kinase 2 [17-19] and SPD-2 [20], have been reported. The phenotype termed centriole overduplication (production of multiple daughter centrioles arranged around each parental centriole) was observed when a positive regulator of centriole duplication, for instance Polo-like kinase-4, is overexpressed [16] due to a disordered mechanism to limit the number of progenitor centrioles. The supernumerary centrosome formation described in Figure 7 is distinct from this phenotype, and therefore, the ARHGEF10-RhoA pathway may not be responsible for the copy number control of procentrioles duplicated from a single parental centriole.

One major mechanism to limit centriole duplication to once in every cell division cycle involves a protein-digesting enzyme termed separase, which is also responsible for sister chromatid separation [21,22]. In the normal cell division cycle, centrioles are prevented from duplicating again during late S and G2 phases, being tightly connected or engaged. At the end of mitosis, the engaged centriole pair becomes dissociated, losing their strict orthogonal orientation. This process, called disengagement, is triggered by the action of separase, and is prerequisite for a new round of centriole duplication [21,22]. Thus, intrinsic block to centriole reduplication throughout the cell cycle and timely activation of separase represent a mechanism to ensure that a new round of duplication can occur only after passage through M phase. Although speculative, disruption of the ARHGEF10-RhoA-dependent signaling pathway by knocking down its component may permit an excess cycle of centriole duplication if sustained engagement of centrioles or suppression of separase activity during late S and G2 phases requires this pathway. The observation that only two among three centrosomes in ARHGEF10-knockdown cells exhibit diminished ARHGEF10 expression (Figure 2C) may be consistent with the idea that only a centrosome that lacks ARHGEF10 undergoes an excess duplication cycle before mitosis.

Multiple mitotic spindles were also observed in cells deficient in the Polo-like kinase-1 target Kizuna [23]. Unlike Kizuna-depleted multipolar cells, however, we did not detect fragmentation of the PCM in ARHGEF10-knockdown cells, suggesting that the ARHGEF10-RhoA pathway is not implicated in stabilization of the PCM in early mitotic phase.

In this paper, we further identified the motor protein KIF3B as a binding partner of ARHGEF10 (Figure 5). KIF3B has been implicated in a variety of physiological responses, including determination of left-right asymmetry [24], axonal transport [25], assembly and maintenance of the excitation-contraction-coupling membranes in skeletal muscle [26], cytokinesis [27] and glucose transport in adipocytes [28]. We observed multiple mitotic spindle poles in KIF3B-knockdown cells (Figure 6), resembling phenotypes observed following expression of a dominant-negative mutant of KIF3B [29]. Considering that ARHGEF10 associates with KIF3B in the centrosome, KIF3B may regulate spindle pole formation through the ARHGEF10-RhoA pathway. KIF3B knockdown may also cause supernumerary centrosomes through cytokinesis defects independently of ARHGEF10 because a dominant-negative mutant of KIF3B caused chromosomal aneuploidy [29]. The mechanisms may be more complicated because it is reported that silencing of another motor protein KIFC5A causes centrosome amplification primarily through reduplication and partly as a result of defects incytokinesis [30].

## Conclusion

ARHGEF10 is a RhoA GEF, which is localized in the centrosome in both G1/S and M phases. ARHGEF10 regulates mitotic spindle formation through the action of RhoA. ARHGEF10-regulaed RhoA may be implicated in the control of centrosome duplication rather than cytokinesis. The motor protein KIF3B is a binding partner of ARHGEF10, a subset of which is co-localized with ARHGEF10 in the centrosome.

Thus, KIF3B may regulate ARHGEF10 upstream of RhoA, being involved in the regulation of centrosome duplication.

## Authors' contributions

TA carried out all the experiments in this study. SU provided experimental materials and participated in discussion. TK provided many useful suggestions and helped to prepare the manuscript. TS supervised the study and drafted the manuscript. All authors read and approved the final manuscript.
